# Implementing subtype‐specific pre‐clinical models of breast cancer to study pre‐treatment aspirin effects

**DOI:** 10.1002/cam4.4756

**Published:** 2022-04-17

**Authors:** Ian S. Miller, Sonja Khan, Liam P. Shiels, Sudipto Das, Alice C. O' Farrell, Kate Connor, Adam Lafferty, Bruce Moran, Claudio Isella, Paul Loadman, Emer Conroy, Susan Cohrs, Roger Schibli, Robert S. Kerbel, William M. Gallagher, Elisabetta Marangoni, Kathleen Bennett, Darran P. O' Connor, Róisín M. Dwyer, Annette T. Byrne

**Affiliations:** ^1^ Department of Physiology and Medical Physics Royal College of Surgeons in Ireland, St Stephens Green Dublin Ireland; ^2^ National Preclinical Imaging Centre Royal College of Surgeons in Ireland, St Stephens Green Dublin Ireland; ^3^ Discipline of Surgery The Lambe Institute for Translational Research, National University of Ireland Galway Galway Ireland; ^4^ School of Pharmacy and Biomedical Sciences Royal College of Surgeons in Ireland, St Stepehen's Green Dublin Ireland; ^5^ UCD School of Bimolecular and Biomedical Science University College Dublin Dublin Ireland; ^6^ Institute for Cancer Research and Treatment University of Turin Turin Italy; ^7^ School of Pharmacy and Medical Sciences, University of Bradford Bradford UK; ^8^ Center for Radiopharmaceutical Sciences Paul Scherrer Institute Villigen Switzerland; ^9^ Sunnybrook Research Institute University of Toronto Ontario Canada; ^10^ Translational Research Department Institute Curie, PSL Research University Paris France; ^11^ Division of Population Health Science Royal College of Surgeons in Ireland Dublin Ireland

**Keywords:** aspirin, breast cancer, cancer prevention lymphangiogenesis, mouse model

## Abstract

**Backgorund:**

Prior data suggest pre‐diagnostic aspirin use impacts breast tumour biology and patient outcome. Here, we employed faithful surgical resection models of HER2+ and triple‐negative breast cancer (TNBC), to study outcome and response mechanisms across breast cancer subtypes.

**Method:**

NOD/SCID mice were implanted with HER2+ MDA‐MB‐231/LN/2‐4/H2N, trastuzumab‐resistant HER2+ HCC1954 or a TNBC patient‐derived xenograft (PDX). A daily low‐dose aspirin regimen commenced until primary tumours reached ~250 mm^3^ and subsequently resected. MDA‐MB‐231/LN/2‐4/H2N mice were monitored for metastasis utilising imaging. To interrogate the survival benefit of pre‐treatment aspirin, 3 weeks post‐resection, HCC1954/TNBC animals received standard‐of‐care (SOC) chemotherapy for 6 weeks. Primary tumour response to aspirin was interrogated using immunohistochemistry.

**Results:**

Aspirin delayed time to metastasis in MDA‐MB‐231/LN/2‐4/H2N xenografts and decreased growth of HER2^+^/TNBC primary tumours. Lymphangiogenic factors and lymph vessels number were decreased in HER2^+^ tumours. However, no survival benefit was seen in aspirin pre‐treated animals (HCC1954/TNBC) that further received adjuvant SOC, compared with animals treated with SOC alone. In an effort to study mechanisms responsible for the observed reduction in lymphangiogenesis in HER2^+^ BC we utilised an in vitro co‐culture system of HCC1954 tumour cells and mesenchymal stromal cells (MSC). Aspirin abrogated the secretion of VEGF‐C in MSCs and also decreased the lymph/angiogenic potential of the MSCs and HCC1954 by tubule formation assay. Furthermore, aspirin decreased the secretion of uPA in HCC1954 cells potentially diminishing its metastatic capability.

**Conclusion:**

Our data employing clinically relevant models demonstrate that aspirin alters breast tumour biology. However, aspirin may not represent a robust chemo‐preventative agent in the HER2^+^ or TNBC setting.

## Graphical abstract

The impact of aspirin as a chemo‐preventative has been under increasing scrutiny as time has passed. Here we present a study detailing the relatively minor impact of aspirin in breast cancer prevention and survival. While Aspirin has effects on the outgrowth of lymph vessels it does not improve overall survival in clinically relevant mouse models of HER2 and triple negative breast cancer.

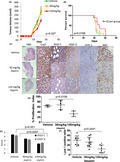

## 1 INTRODUCTION

Aspirin (acetylsalicylic acid) is a common drug used in cardiovascular disease prevention and pain control. It has emerged as a promising agent for the chemoprevention of certain cancers, with several clinical and epidemiological studies providing supporting evidence, particularly with respect to gastrointestinal tumours.^1^ Aspirin (an irreversible inhibitor of both cyclooxygenase 1 and 2 (COX‐1 and COX‐2) has also been proposed as a putative chemopreventative agent in the breast cancer (BC) setting, where COX‐2 has been shown to expressed in up to 40% of BCs,^2^ and is associated with larger tumour size, higher histological staged tumours, negative hormone receptor status, a high proliferation rate, and HER2 amplification.^3^ Moreover, women with tumours that express COX‐2 are also more likely to present with positive lymph nodes (LN) at diagnosis and to die from BC.^4^ Several pre‐clinical and epidemiological studies^5^, ^7^ have suggested that pre‐diagnostic drug exposures can have significant effects on breast tumour biology at time of diagnosis. Epidemiological studies have also reported that aspirin use prior to BC diagnosis is associated with a significant reduction in the incidence of LN metastasis.^8^, ^9^ The use of pre‐diagnostic aspirin has also been linked with improved survival, with the strongest association found in patients with HER2+ BC.^6^

In general, the molecular mechanisms underlying low‐dose aspirin effects in tumours are still largely unknown^10^ and have been highlighted by the National Cancer Institute's ‘Provocative Questions In Cancer’ initiative as a key outstanding question.^11^ Several studies have investigated the preventative role of aspirin in BC, with most focusing on aspirin‘s ability to block metastasis via inhibition of lymphangiogenesis. Pre‐clinical experiments have shown that overexpression of the lymphangiogenic factors VEGF‐C/‐D by Her2+ and TN breast tumours inhibits prostaglandin degradation and induces lymphatic hyperplasia and increases LN metastasis.^8^, ^9^, ^12^ In this context, low‐dose aspirin has been shown to inhibit COX‐2 activity in TN tumours and reduce lymphangiogenesis via the VEGF‐C/‐D axis.^12^ Furthermore, Johnson et al. have studied the antiplatelet activity of aspirin regarding BC metastasis (Her2^+^ and TN). It was found that low‐dose aspirin inhibits the release of metastasis‐promoting platelet‐derived factors into the tumour microenvironment.^13^ It has also been previously reported that low‐dose aspirin can have a direct effect on the tumour microenvironment and stroma, and may play a role in tumour control and metastasis.^14^, ^15^ Su et al. demonstrated that inhibition of the tumour stroma by aspirin may be one potential mechanism to inhibit carcinogenesis and tumour spread. Specifically, it was shown that aberrant activation of the tumour microenvironment allowed the release of several angiogenesis regulators. It was therefore suggested that aspirin may restore the balance of pro‐ and anti‐angiogenic factors to ‘normalise’ tumour vasculature and (to some extent) shape the tumour microenvironment to diminish tumour aggressiveness and progression, and enhance the therapeutic response.^15^

Herein, we sought to mechanistically interrogare the effect of pre‐treatment aspirin as a potential chemo‐preventative approach across BC subtypes. Specifically, we have ‘reverse translated’ a clinically relevant low‐dose aspirin exposure protocol in subtype‐specific mouse models which have previously been shown to accurately predict the outcome of clinical trials for targeted therapies delivered in the metastatic, adjuvant or neoadjuvant setting.^16^, ^17^, ^18^ Employing these models, we have incorporated surgical resection of the primary tumour and treatment with chemotherapy to further study aspirin chemo‐preventative effects. We also employed the HCC1954 trastuzumab‐resistant cell line (due to a gain in function mutation in PI3K [H1047R]),^19^ to assess whether aspirin pre‐treatment could overcome trastuzumab resistance. Finally, to study tumour microenvironment effects of aspirin therapy we utilised a co‐culture system to assess the impact of aspirin on secretion of lymphangiogenic factors.

## 2 METHODS

### 2.1 Animal studies

In vivo studies were performed at University College Dublin (UCD) and were approved by UCD Animal Research Ethics Committee (AREC). All work was performed according to local rules and regulations and under Health Products Regulatory Authority HPRA licence number AE18982/P039. Sample sizes were calculated based on a Z‐test with a Bonferroni correction for multiple testing at a power of 80%. Mice were purchased from an approved supplier (Charles River UK), and were aged between 4 and 6 weeks, weight range 20–25 g. They were housed in individually ventilated cages, provided with suitable environmental enrichment including plastic shelters and paper nesting material, given food and water ad libitum and maintained under a 12 h dark/light cycle. All thereputic doses were based on previously published data and were shown to be well tolerated.^18^, ^20^ Animals were euthanised once they reached ethical endpoint (tumour reached 15 mm diameter, 20% weight loss or an overall decline in animal well‐being).

### 2.2 MDA‐MB‐231/LN/2‐4.H2N Model

MDA‐MB‐231/LN/2‐4/H2N cells (5 × 10^5^) were orthotopically implanted into the right inguinal mammary fat pad of female non‐obese diabetic severe combined immunodeficient (NOD/SCID: NOD.CB17‐Prkdcscid/NcrCrl) mice (*N* = 30, 20–25 g 10 per group) under anaesthesia (Isoflurane induction 4%, maintenance 1.5% and 0.8 L O_2_/min). Forty‐eight hours after implantation of tumour cells animals were randomised and commenced on an aspirin pre‐treatment regimen. Animals either received 100 μl vehicle (water) (*N* = 10), 30 mg/kg aspirin (*N* = 10) or 120 mg/kg aspirin (*N* = 10) PO daily until tumours reached 250 mm^3^ (Figure S1E). Aspirin therapy was withdrawn 48 h prior to surgical removal of the primary tumour to minimise bleeding. Resection of tumours was performed as previously described.^21^, ^22^ Tumours and metastatic dissemination were monitored by BLI. The methods for BLI are found in Data S1.

### 2.3 Trastuzumab‐resistant Her2^+^ HCC1954 model

HCC1954_Luc2 (10 × 10^6^) were orthotopically implanted and treated as above until tumours reached ~250 mm^3^. Resection of tumours was performed as above. Three weeks following resection mice were treated with either HER2_SOC [paclitaxel (5 mg/kg) + the HER2 targeting monoclonal antibody trastuzumab (loading dose 15 mg/kg then 10 mg/kg)], or Vehicle control (0.5% DMSO in PBS) once per week for 6 weeks (*N* = 12 per group). All mice which had received aspirin therapy prior to resection were treated with SOC (Figure S1F). Tumour growth was measured via calliper assessment of primary tumour regrowth (Volume calculated using the formula [LxW^2^] x0.5).

### 2.4 Enzyme‐Linked Immunosorbent Assay (ELISA) quantification of VEGF‐C in conditioned media from MSC +/− HCC1954

VEGF‐C was quantified in conditioned medium (CM) from MSCs +/− HCC1954 using Quantikine® ELISA (R&D Systems, England, #DVEC00), according to the manufacturer's instructions. Absorbance was measured at 450 nm using a plate reader (Multiskan Ascent; Thermo Fischer Scientific, Germany, #A51119500C). A standard curve was prepared by plotting absorbance values against concentrations of standards. The concentration of unknowns was determined based on this curve. Each measurement was performed in duplicate and the mean values of triplicate experiments were employed for statistical analysis.

### 2.5 Tubule formation assay

CM was collected from MSCs cultured alone and MSCs co‐cultured with HCC1954 cells in the presence or absence of Aspirin (2.5 mM or 7.5 mM). Human umbilical vein endothelial cells (HUVECs) were resuspended in the CM (2.5 × 10^5^ cells/ml), and were seeded onto Growth Factor Reduced Matrigel (ThermoFischer Scientific, #11523550), incubated at 37°C for 4–6 h, and tubule formation imaged using an inverted phase contrast microscope.^23^ Image analysis was performed using the angiogenesis analyser module of ImageJ (https://imagej.nih.gov/ij/), with the total segment length per image quantified.

### 2.6 Human angiogenesis array assay

HCC1954 Her2+ cells were lysed with Triton‐X lysis buffer with 10 μl/ml Protease inhibitor cocktail (Fisher Scientific). Protein quantification was performed using Pierce™ BCA protein Assay (ThermoFischer Scientific,#10,678,484). Analysis of proteins by Proteome Profiler™ (Human Angiogenesis Array, R&D Systems®, #ARY007) was performed following the manufacturer's instructions. Briefly, 300 μg of protein lysate from HCC1954 alone or HCC1954 + 7.5 mM Aspirin were incubated with an antibody cocktail for 1 h at room temperature. Protein–antibody mix were then incubated with each membrane at 4°C overnight. The membranes were then incubated with streptavidin‐HRP for 30 min and chemiluminescence visualised using the Chemidoc (Bio‐Rad, #12003153). Mean pixel density of each duplicate dot was determined using ImageLab software. Reference spots on each membrane supported normalisation of variance between membranes.

### 2.7 Patient‐derived Xenograft (PDX) model of triple‐negative breast cancer

The TN PDX (designated HBCx‐12A), was derived from a primary tumour resection specimen from a Caucasian female who received neoadjuvant docetaxel. Ethical approval for the use of the tissue was sought from the Institut Curie Hospital committee and by informed consent from the patient. Fragments of tumour HBCx‐12a were grafted into the intrascapular fat pad of *N* = 2 female NOD/SCID, as previously described^24^ and then further expanded in *N* = 8 mice.^24^ After tumour expansion, tumour fragments were implanted into *N* = 48 (20‐25 g) NOD/SCID mice. Forty‐eight hours after tumour implantation mice were dosed by oral gavage as above. Tumours were allowed to develop until they reached ~250 mm. Tumours were resected as previously described.^18^ After 3 weeks all mice except vehicle (true vehicle: i.e., *N* = 12 mice did not receive SOC or aspirin) mice began 6 weeks of weekly 5 mg/kg paclitaxel (Figure S1F). Mice were monitored closely until morbidity, or 200 days (post tumour implantation), when all animals were euthanized by cervical dislocation.

### 2.8 DAB‐mediated immunohistochemistry

A minimum of three tumours per treatment group were selected for immunohistochemical (IHC) analysis using DAB probes for Ki67 (1:150 rabbit polyclonal, Milipore, AB9260), COX‐2 (1:100 rabbit polyclonal Abcam,AB15191), VEGF‐C (1:50 mouse monoclonal Abcam,AB106512) VEGF‐D (1:50 rabbit monoclonal Abcam, 155,288), LYVE1 (1:150 rabbit polyclonal, Abcam, AB281587) and pancytokeratin ae1/3 (1:100 mouse monoclonal Abcam, 27,988). Briefly, extracted tumours were fixed in 10% formalin (Sigma Aldrich, HT501128) for a minimum of 24 h and were then processed overnight in an automatic tissue processor (Lecia TP1020). Samples were then paraffin embedded and 5 μm sections cut. Sections were deparaffinised and subsequently probed using the Lab Vision UltraVision LP polymer Detection System (Thermo Scientific, #10596014). Sections were blocked for 10 mins with 3% hydrogen peroxide (Sigma Aldrich, 88,597) followed by Ultra Vision Block 10 mins. Antibodies diluted ion PBS were then incubated for 1 h at room temp. Antibodies were then washed off with PBS and Ultra Vision primary antibody enhancer added for 15 mins, followed by ultra vision AP polymer for a further 15 mins. Finally DAB Quanto (Thermo Scientific, #12693967) was added and incubated for 10 mins. Images were taken using Zeiss Axiovert microscope at 10x magnification. Ki67 images were analysed by colour deconvolution in ImageJ and counting all positive brown nuclei and dividing by the total number of blue nuclei (positive/total *100). LYVE 1 images were analysed by applying a 15,000 pixel^2^ grid over the image in ImageJ and counting the number of times positive vessels cross the grid. VEGF‐C/‐D probed sections were scored semi‐quantitatively by two independent observers using the Allred Score Method based on proportion of positive cells (scored on a scale of 0–5) and staining intensity (scored on a scale of 0–3). The proportion and intensity were then summed and averaged to produce a total score where 0–2 was finally regarded as negative while 3–8 as positive.^25^

### 2.9 RT‐qPCR analysis of beta‐catenin

PCR was perfomed on an Applied Biosystems 7900HT thermocycler according to the manufacturer’s instructions. RNA was extracted from primary resected HCC1954 tumours (3 per group) using the Qiagen RNEasy extraction kit according to the manufacturer's instructions. The integrity of the RNA was measured on an Agilent 2100 bioanalyzer nano‐chip and samples with a RIN greater than 1.7 were converted to cDNA (1 μg––Superscript IV First strand synthesis system oligo‐DT Invitrogen) according to the manufactures instructions. Subsequently 1 μl of cDNA was subjected to qPCR. The primers for qPCR were self‐designed (www.Primer3.ut.ee), and commercially synthesised by Sigma. Standard RT‐ qPCR (Sybr Green Master mix; Life Technologies) was used (max 40 cycles) to measure the expression of ctnnb1 with melt curve analysis to ensure primer optimization (Annealing temperature for primers:60°C. Range of CT values obtained for beta‐catenin 31.01–32.05. Range of CT values for RPLPO 20.03–20.27). Each sample and no template control were measured in triplicate. Relative expression was based on the 2^‐ΔΔCT^ method. Expression was normalised to the expression of the housekeeper rplpo.

Primers *ctnnb1* 5′: GGCCATGGAACCAGACAGAA 3′: ACCCTCTGAGCTCGAGTCAT.

Primers *rplp0* 5′: CTGGAGAAACTGCTGCCTCA 3′: CAATGGTGCCCCTGGAGATT.

### 2.10 Statistical analyses

Area under the curve analysis (AUC) of the plasma curves was performed to determine the kinetics of salicylate absorption in the plasma. For statistical comparison, Mann–Whitney and Kruskal–Wallis tests performed for nonparametric in vivo data and used to determine significance in IHC. Log‐rank tests for survival time data. Statistical analysis for the in vivo study was performed using GraphPad Prism 7 (La Jolla, CA, USA). *P* values ≤0.05 were considered statistically significant. All replicates shown are biological replicates.

## 3 RESULTS

### 3.1 Pharmacokinetic analysis of aspirin to establish clinically relevant dosing regimen for aspirin in mice

As aspirin is rapidly metabolised to salicylate, we determined the kinetics of salicylate absorption (7.5 mg/kg and 30 mg/kg) in plasma over time (Figure S1A). The AUC for 7.5 mg/kg aspirin was 7.9 μg*h/ml and the AUC for 30 mg/kg was 24.3 μg*h/ml (Figure S1B). As the absorption kinetics were shown to be linear (*R* ^2^ = 0.9559 Figure S1C) we correlated mouse values with human values (19–21) and extrapolated a dose in mice equivalent to the human low dose. A standard oral 75 mg a day dose of aspirin correlated with a dose of 30 mg/kg in mice (Figure S1D). Furthermore, a dose of 300 mg a day in humans correlated with a dose of 120 mg/kg in mice (Figure S1D).

### 3.2 Aspirin pre‐treatment delays primary tumour growth and metastatic dissemination of Her2^+^ MDA‐MB‐231/LM2‐4/H2N tumours but does not increase overall survival compared to SOC in the trastuzumab‐resistant HCC1954 Her2^+^ BC model

#### 3.2.1 MDA‐MB‐231/LM2‐4/H2N_Luc2 HER2 ^+^ model

**FIGURE 1:**
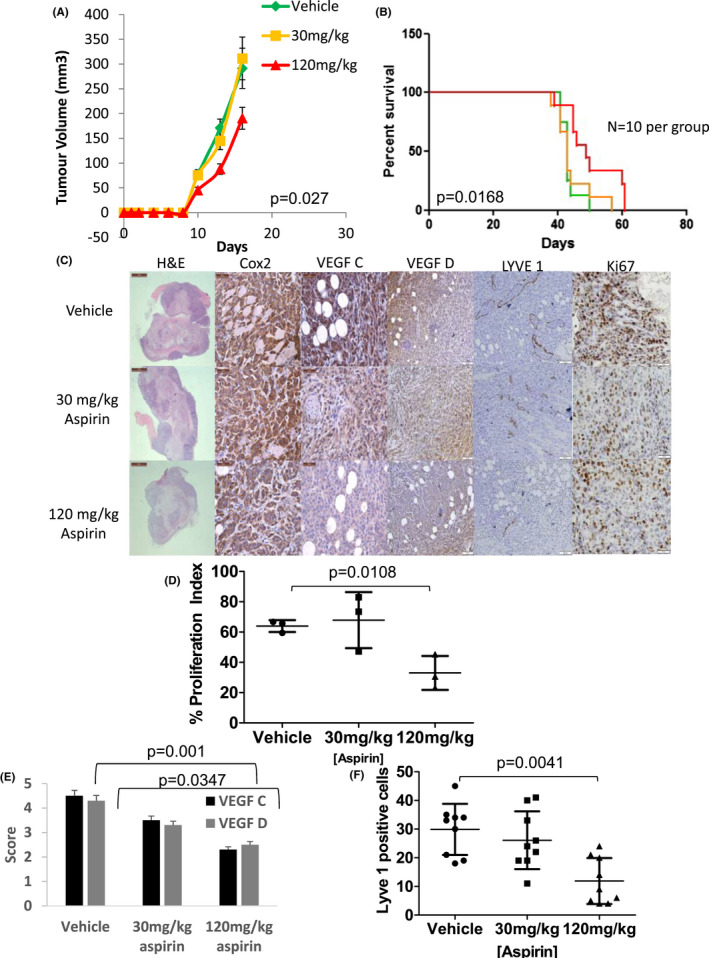
Aspirin reduces the growth of primary tumours, increases time to metastasis and reduces lymphangiogenesis via a reduction in lymphangiogenic factors in the MDA‐MB 231/LN2‐4/H2N orthotopic cell line model of Her2^+^ BC. (A) Graph displaying the growth of primary tumours while treated with aspirin. Aspirin of 120 mg/kg daily shows a significantly smaller tumour at time of tumour resection (*p* = 0.027 *N* = 10 Mann–Whitney, error bars represent standard error of the mean). (B) Kaplan–Meier curve of time to metastatic progression measured in days post implantation of tumour cells. Animals treated with 120 mg/kg of aspirin had a significant delay in time to metastasis (*p* = 0.0168 *N* = 10 log rank test). (C) Representative micrographs (100X) of primary MDA‐MB‐231 LN2‐4/H2N cell line xenografts stained with H&E or DAB probe for cell inflammatory marker COX‐2, the lymphangiogenic markers VEGF‐C and VEGF‐D the lymphatic vessel marker LYVE1 and the proliferation marker Ki67. (D) Analysis of Ki67‐based proliferation. Tumours treated with 120 mg/kg aspirin display significantly lower (*p* = 0.0108 *N* = 3, Kruskal–Wallis) proliferation index compared to vehicle or 30 mg/kg aspirin‐treated tumours. (E) Analysis of the lymphangiogenic markers VEGF‐C and –D. Tumours treated with 120 mg/kg aspirin show significantly lower expression of the two markers (*p* = 0.001 and 0.0347 Mann–Whitney). (F) Analysis of the lymphatic vessel marker Lyve1. Tumours treated with 120 mg/kg aspirin display significantly fewer lymph vessels compared to 30 mg/kg or vehicle (*p* = 0.0041 *N* = 3 Kruskal–Wallis)‐treated tumours. Error bars represent standard deviation (SD) *N* = minimum 3 tumours per group and four images per tumour

Animals treated with 120 mg/kg aspirin daily had significantly smaller tumours at time of surgical resection (*p* = 0.0270 Students *t*‐test *N* = 10) compared to other groups. BLI (Figure S2A) confirmed efficient excision of tumour. Mice were monitored and continued to be imaged weekly until the appearance of detectable metastases by BLI whereupon animals were euthanized (Figure S2B). N.B Due to concerns for animal welfare, first appearance of distant metastasis was the study endpoint (i.e., Time to Progression [TTP]). This was required by the University College Dublin Animal Research Ethics Committee (AREC). During the experiment, most animals (vehicle 10/10 (100%), 30 mg/kg 10/10 (100%) and 120 mg/kg aspirin 8/9 (89%)) displayed primary tumour regrowth on BLI (Figure S2A,C). However, there was a significant (*p* = 0.00265 *N* = 10 Mann Whitney nonparametric test) delay in the regrowth of tumours (TTP) treated with 120 mg/kg of aspirin. Furthermore, there was a significant delay (*p* = 0.0168 *N* = 10 Log‐rank test) in the appearance of metastases in mice treated (120 mg/kg aspirin daily) prior to tumour resection compared to vehicle and 30 mg/kg aspirin‐treated animals (Figure 1B). The median time to appearance of metastases in both vehicle and 30 mg/kg aspirin cohorts was 43 days. The median time to metastases in the 120 mg/kg aspirin group was 49 days. Following euthanasia, organs and LNs were removed an imaged ex vivo. Figure S2D shows that aspirin treatment did not alter the pattern of metastatic dissemination.

IHC analysis of primary resected tumours was carried out to investigate the effect of aspirin pre‐treatment on proliferation, necrosis and lymphangiogenesis. Neither 30 mg/kg nor 120 mg/kg daily aspirin (Figure 1C) altered the level of tumour necrosis (assessed by Haematoxylin & Eosin) or the COX‐2 expression. However, tumours treated with 120 mg/kg aspirin daily showed a significant decrease *p* = 0.00328 Mann–Whitney nonparametric test) in proliferation (Figure 1D). There was a significant decrease (*p* = 0.001 and *p* = 0.0347, respectively Mann–Whitney nonparametric test) in the expression of lymphangiogenic factors (VEGF‐C/‐D) when treated with 120 mg/kg aspirin daily (Figure 1E). A concomitant decrease (*p* = 0.0057 *N* = 5 Mann Whitney nonparametric test) in the number of primary tumour lymph vessels was also observed in tumours treated with 120 mg/kg of aspirin daily (Figure 1F).

#### 3.2.2 Trastuzumab‐resistant HCC1954_Luc2 HER2+ model

**FIGURE 2:**
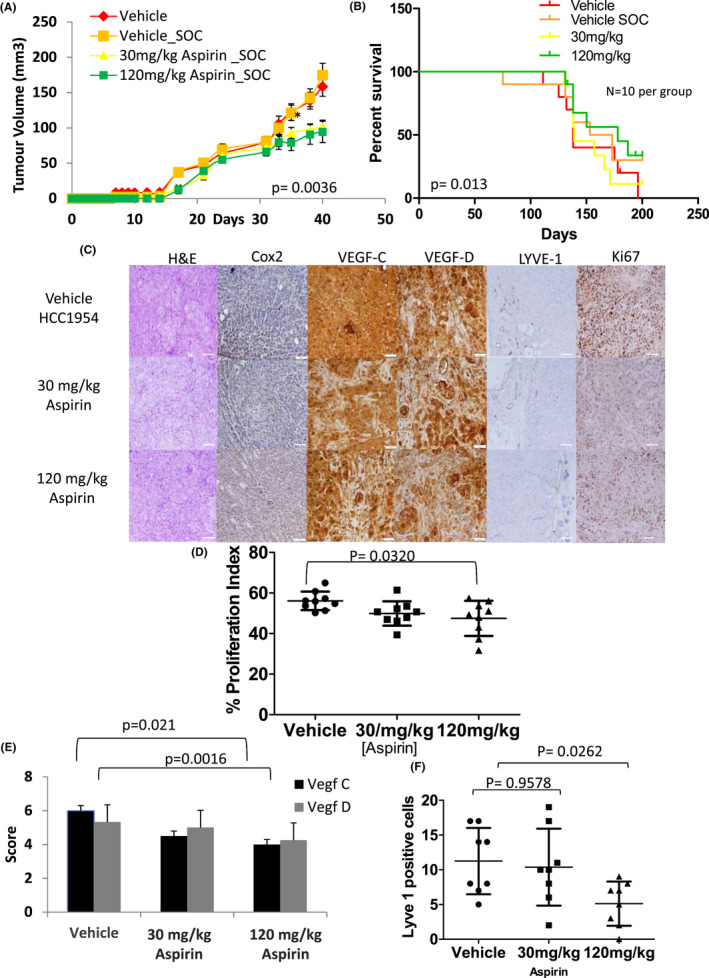
Low‐dose aspirin reduces growth and reduces lymphangiogenesis via a reduction in lymphangiogenic factors in primary tumours but does not improve survival after adjuvant trastuzumab and paclitaxel in an orthotopic trastuzumab‐resistant HCC1954 cell line model of breast cancer. (A) Graph displaying the growth of primary tumours while treated with aspirin. Both 30 mg/kg and 120 mg/kg daily aspirin shows a significantly smaller tumour at time of tumour resection (day 40 post implantation) (*p* = 0.0036 *N* = 12 Kruskal–Wallis, error bars represent standard error of the mean). (B) Kaplan–Meier curve of time to death measured in days post implantation of tumour cells. Animals which died from nontumour‐related causes are censored for survival analysis. There was no significant difference (*p* = 0.103 log rank test median survival vehicle: 138 days, Vehicle SOC: 163 days 30 mg/kg aspirin_SOC:139 days and 120 mg/kg aspirin_SOC: 178 days) between any treatment and vehicle. (C) Representative micrographs (100X) of primary HCC1954 cell line xenografts stained with H&E or DAB probe for COX‐2, VEGF‐C and VEGF‐D, Ki67. Scale bars are 50 μm. (D) Analysis of Ki67‐based proliferation. Tumours treated with 30 mg/kg aspirin (*N* = 3) and 120 mg/kg aspirin (*N* = 3) display significantly (*p* = 0.0320 Kruskal–Wallis) lower proliferation index compared to vehicle‐treated tumours. (E) Analysis of the expression of lymphangiogenic markers VEGF‐C and –D. Tumours treated with both 30 mg/kg and 120 mg/kg aspirin shows significantly lower expression of VEGF‐C (*P* = 0.0210 and *p* = 0.0016, respectively *N* = 3 Mann–Whitney). No difference in VEGF‐D expression is noted (*p* = 0.8221 and *p* = 0.2078 *N* = 3). (F) Analysis of the lymphatic vessel marker Lyve1. Tumours treated with 120 mg/kg aspirin display significantly (*p* = 0.0262 Mann–Whitney two tailed test *N* = 3) fewer lymph vessel compared to 30 mg/kg (*p* = 0.9578) or vehicle–treated tumours. Error bars represent standard deviation (SD). *N* = minimum three tumours per group and four images per tumour

It has previously been shown that the HCC1954 cell line has a gain‐of‐function hot spot mutation (H1047R, PI3K gain‐of‐function) which underpins resistance to trastuzumab‐mediated HER2 blockade (Figure S3).^19^ We therefore employed this model to assess the ability of pre‐treatment aspirin to overcome trastuzumab resistance and resensitise the tumour to SOC therapy. Tumours treated with 30 mg/kg and 120 mg/kg aspirin grew at a significantly slower rate (*p* = 0.0036 *N* = 12 per group Kruskal–Wallis nonparametric test) than tumours in the vehicle cohort (Figure 2A) and were smaller at time of resection. Mice were subsequently treated with SOC (trastuzumab and paclitaxel) once a week for 6 weeks, and then monitored until they reached morbidity. After 200 days several animals were still alive (Vehicle SOC = 5, 30 mg/kg aspirin = 1, 120 mg/kg = 6 Figure 2B). At this time point, all remaining mice were euthanized and censored for survival analysis. Mice pre‐treated with low‐dose aspirin (either 30 mg/kg or 120 mg/kg aspirin daily) prior to tumour resection followed by adjuvant SOC received no significant survival benefit (Figure 2B) compared to mice who received no aspirin prior to resection and adjuvant SOC (*p* = 0.130, *N* = 12 per group, median survival Vehicle_vehicle = 138, Vehicle_SOC = 163 30 mg/kg aspirin_SOC = 139,120 mg/kg aspirin_SOC = 178 Figure 2B Log rank test). It was noted, that no significant increase in survival was observed in animals that received adjuvant SOC (but no aspirin prior to resection) compared to animals that neither received pre‐treatment aspirin nor adjuvant SOC (*p* = 0.526 *N* = 12 per group). (Although planned, BLI was not implemented due to an unforeseen closure of the small animal imaging facility).

IHC was used to determine the effect of aspirin pre‐treatment on proliferation, necrosis and lymphangiogenesis. Congruent with observations in the MDA‐MB‐231/LM2‐4/H2N _Luc2 model there was no change in the extent of necrosis or in the expression of COX‐2 (Figure 2C). Nevertheless, tumours treated with 30 mg/kg and 120 mg/kg aspirin daily both demonstrated a significantly (*p* = 0.002 and 0.014, respectively Mann–Whitney nonparametric test) reduced proliferation index compared to the vehicle (56% vs. 49% vs. 47%, Figure 2C,D). A decrease in VEGF‐C/‐D expression was also evident in the HCC1954 model (Figure 2E) (VEGF‐C 30 mg/kg *p* = 0.021120 mg/kg *p* = 0.0016 *N* = 4 VEGF‐D 30 mg/kg aspirin *p* = 0.103, 120 mg/kg aspirin *p* = 0.033 *N* = 4). Again, a concomitant decrease in the number of primary tumour lymph vessels was also observed in tumours treated with 120 mg/kg of aspirin (Figure 2F *p* = 0.0411 Mann–Whitney nonparametric test).

### 3.3 Aspirin alters the secretome of Her2^+^ HCC1954 cells and abrogates the secretion of the pro‐lymphangiogenic factor VEGFC by MSC in co‐culture with HCC1954

#### 3.3.1 VEGF‐C secretion

**FIGURE 3:**
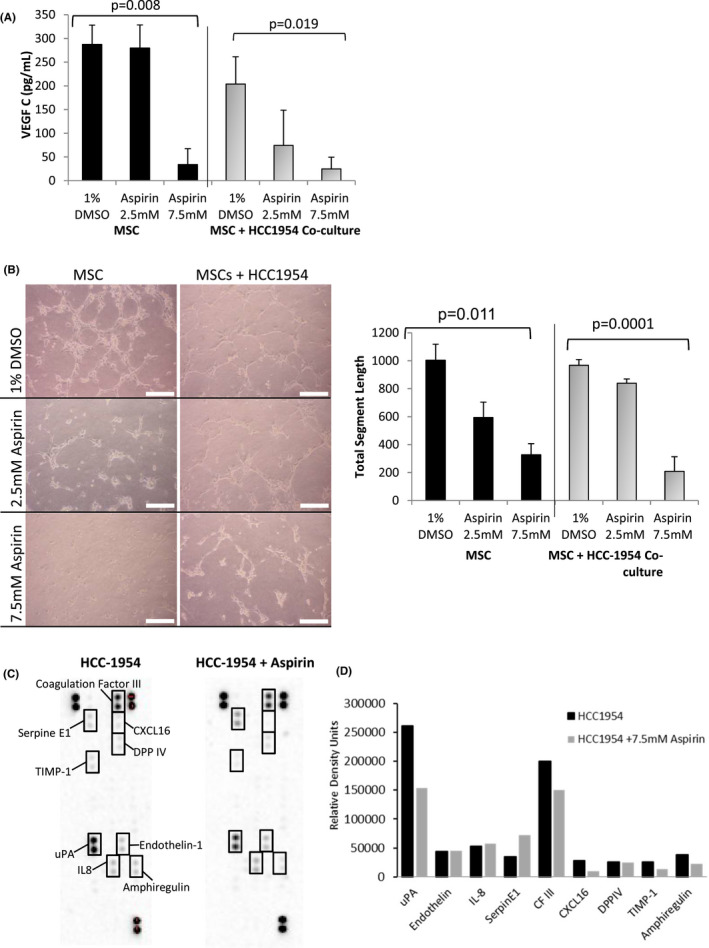
Aspirin alters the secretome of HCC1954 cells and abrogates the secretion of the pro‐lymphangiogenic factor VEGFC by MSCs in co‐culture with HCC1954. (A) Graph displaying the secretion of VEGF‐C by MSC or MSC in co‐culture with HCC1954 tumour cells as determined by ELISA. MSCs secrete significant amounts of VEGF‐C both alone and in co‐culture with HCC1954. Aspirin of 7.5 mM in MSCs and MSC in co‐culture significantly diminishes the secretion of VEGF (*p* = 0.008 and 0.019 respectively, *N* = 3 One‐way Anova). Aspirin of 2.5 mM had minimal effect in either condition. (B) Micrographs and graphs displaying the effect of aspirin on tubule formation. HUVEC cells were treated with conditioned media from MSCs or MSCs in co‐culture with HCC1954 with or without aspirin (2.5 mM and 7.5 mM). Both MSCs alone and MSCS in co‐culture with HCC1954 promote the formation of HUVEC tubules. A small but not significant decrease in tubule formation is evident when treated with 2.5 mM aspirin. Aspirin of 7.5 mM significantly diminishes (*p* = 0.011 and 0.0001 *N* = 3 One‐way Anova) tubule formation in HUVECs treated with MSC or MSC co‐cultured with HCC1954 conditioned media. (C) Expression of pro‐angiogenic factors in HCC‐1954 BC cells was also assessed using a human angiogenic protein array in the presence and absence of aspirin (7.5 mM asprin only). A significant decrease in uPA (−42%) was determined with a concurrent increase in the uPA inhibitor Serpin E1. No other factors were significantly altered. Error bars represent +/− standard error of the mean

To study the effect on aspirin on stromal tissue, primary MSC were cultured alone or co‐cultured with trastuzumab‐resistant HCC1954 cells and treated with 2.5 mM and 7.5 mM aspirin. CM was harvested and changes in VEGF‐C secretion were measured using ELISA (Figure 3A). MSCs secreted the high levels of VEGF‐C (305 ± 35 pg/ml) (Figure 3A). VEGF‐C secretion was significantly (*p* = 0.0080 *N* = 3 Mann–Whitney nonparametric test) abrogated in cell conditioned media of MSC following of addition 7.5 mM of aspirin (34 ± 32 pg/ml). Aspirin of 2.5 mM did not significantly diminish the secretion of VEGF‐C in MSC alone (280 ± 42 pg/ml). The co‐culture of HCC cells + MSC resulted in secretion of VEGF‐C (204 ± 52 pg/ml). The addition of 7.5 mM aspirin significantly (*p* = 0.019 *N* = 3) reduced the secretion of VEGF‐C. Aspirin of 2.5 mM showed no reduction (0.074 *N* = 3 Mann–Whitney nonparametric test) of VEGF‐C secretion.

#### 3.3.2 Aspirin inhibits MSC conditioned media‐driven HUVEC tubule formation

MSCs were shown to stimulation HUVEC tubule formation in vitro (Figure 3B, 1004 ± 153 pixels). The addition of 2.5 mM and 7.5 mM aspirin showed a significant (one‐way ANOVA *p* = 0.011) dose‐dependant reduction in tota vessel segment length. Tubule formation was as prominent (968 ± 35 pixels) in response to conditioned media harvested from HCC cells co‐cultured with MSCs. The addition of 7.5 mM of aspirin to HCC cells co‐cultured with MSCs showed a significant (*p* = 0.0001 Mann–Whitney nonparametric test) disruption in angiogenesis (209 ± 104 pixels). However, the addition of 2.5 mM of aspirin to HCC cells co‐cultured with MSCs did not alter the tubule formation process (840 ± 31 pixels).

#### 3.3.3 Aspirin alters the secretome of trastusumab resistant HCC1954 Her2^+^ cells in vitro

Expression of pro‐angiogenic factors was assessed by a Human Angiogenesis Array assay, following aspirin exposure in HCC cells (Figure 3C). Alterations in the levels of angiogenesis‐ related proteins were observed, with the greatest decrease seen in Urokinase Plasminogen Activator (uPA, −42% decrease; from RDU: Mean(SEM) 261,538(12,522) to 154,033(4935), *p* = 0.03) and an increase in its inhibitor Serpin E1 (+55% increase; from RDU: 35,116 (2376) to 71,793(5495), *p* = 0.05) (Figure 3D).

### 3.4 Low‐dose aspirin promotes autophagy and beta‐catenin activity in primary trastuzumab‐resistant HCC1954 Her2^+^ tumours in a dose‐dependant manner

**FIGURE 4:**
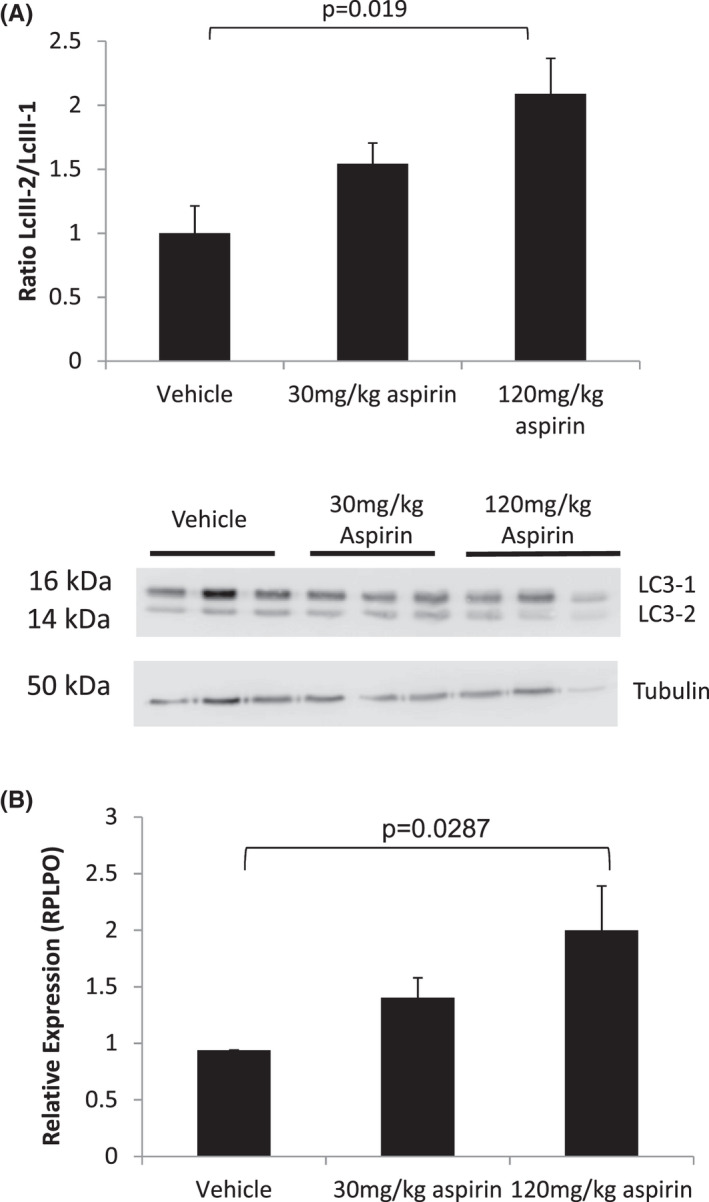
Low‐dose aspirin activates of autophagy and upregulates of beta‐catenin in trastuzumab‐resistant HCC1954 primary tumours. (A) Western blot analysis and subsequent densitometry for the autophagy marker LC3 (a protein involved in autophagosome formation). A twofold increase in LC3‐II (16 kDa) vs. LC3‐I (14Kda) expression in tumours treated with 120 mg/kg asprin a day (*p* = 0.019 *N* = 3 per group Student's *t‐*Test). Shown are original blots. Blots were cut to facilitate probing of different markers on the same blot. B) *Beta‐catenin (ctnnb1)* expression was found to be upregulated by qRT‐PCR in a dose‐dependant manner in resected primary HCC1954 tumours treated aspirin daily. Aspirin of 120 mg/g showed a significant increase in expression relative to housekeeper gene expression (relative expression vs. housekeeper *rplp0 p* = 0.0287 *N* = 3 per group Student's *t‐*test). Error bars represent +/− SEM

To mechanistically assess the ability of aspirin to delay primary tumour growth, we studied the induction of autophagy within a cohort (*N* = 3 per group) of resected primary trastuzumab‐resistant HCC1954 Her2^+^ tumours treated daily with 30 mg/kg aspirin,120 mg/kg aspirin or vehicle. Upon western blot and densitometry analysis (Figure 4A) for cleavage of LC3‐I to LC3‐II we observed a significant (*p* = 0.019 *N* = 3 Student *t‐*test) ~twofold increase in LC3‐I cleavage in tumours treated with 120 mg/kg aspirin daily relative to tubulin expression. The classical WNT pathway marker beta‐catenin (*ctnnb1)* has been shown to negatively regulate autophagy in tumours cells. Upon qRT‐PCR analysis of *ctnnb1* in cohort (*N* = 3 per group) resected primary trastuzumab‐resistant HCC1954 Her2^+^ tumours treated daily with 30 mg/kg aspirin, 120 mg/kg aspirin or vehicle showed a dose‐dependent increase in the expression of *ctnnb1* (Figure 4B). Primary tumours treated with 120 mg/kg displayed a significant (*p* = 0.0287 *N* = 3 Students *t‐*test) upregulation of *ctnnb1* (+1.9979) in tumours aspirin relative to the housekeeping gene *rplpo*.

### 3.5 Low‐dose aspirin pre‐treatment delays TNBC PDX tumour growth but does not improve survival following primary tumour resection and treatment with SOC chemotherapy

**FIGURE 5:**
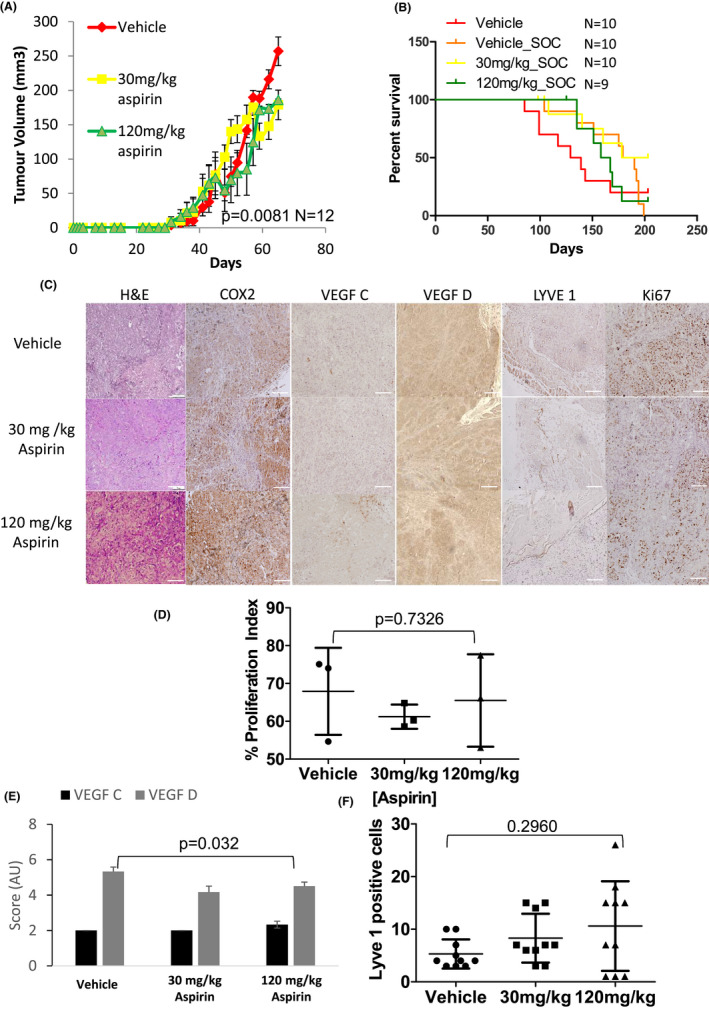
Aspirin delays the growth of primary tumour but does not affect lymphangiogenesis or cell proliferation and does not prolong overall survival compared to paclitaxel alone in a PDX model of TNBC. (A) Graph displaying the growth of primary tumours while treated with aspirin. Both 30 mg/kg and 120 mg/kg daily aspirin shows a significantly smaller tumour at time of tumour resection (day 65 post implantation) (*p* = 0.0081 *N* = 12 Student's *t‐*test). Following tumour resection, mice were treated with SOC (paclitaxel 5 mg/kg) or vehicle once a week for 6 weeks. B) Kaplan–Meier curve of time to death measured in days post implantation of tumour cells. There was no significant delay in survival in any group compared to vehicle_soc (*p* = 0.2451 *n* = 12 log rank test). Animals which died from nontumour‐related causes are censored for survival analysis. (C) Representative micrographs (100X) of primary PDX xenografts stained with DAB probe COX‐2, VEGF‐C and VEGF‐D, LYVE1 and Ki67. Scale bars are 100 μm. (D) Analysis of Ki67‐based proliferation. Aspirin does not alter the proliferation index of the TNBC PDX tumour as detailed by Ki67 expression (*P* = 0.7326 Kruskal–Wallis Test). (E) Analysis of the lymphatic vessel marker Lyve1. No significant difference (*p* = 0.2960) in the number of lymph vessels in any of the treated tumours was evident. Error bars represent standard deviation (SD) *N* = minimum three tumours per group and four images per tumour. (F) Analysis of the expression of lymphangiogenic markers VEGF‐C and ‐D. As determined by the Allred method of IHC scoring, the TNBC PDX expressed very low levels of VEGF‐C and was considered negative for VEGF‐C. VEGF‐D displayed a small but significant decrease in expression (*p* = 0.032 Kruskal–Wallis)

At time of resection, tumours treated with either 30 mg/kg or 120 mg/kg aspirin daily (Figure 5A) were significantly smaller than those not treated with aspirin (*p* = 0.0081 *N* = 12 *Student's t*‐ test). Once the tumour was resected, mice were allowed to recover for 3 weeks and were then treated with SOC (paclitaxel 5 mg/kg IP once per week) for 6 weeks. Mice were then followed to morbidity or for 200 days. Mice pre‐treated with low‐dose aspirin (either 30 mg/kg or 120 mg/kg aspirin) prior to tumour resection and adjuvant SOC, received no significant increase in survival (Figure 5B) compared to mice who did not receive aspirin prior to resection and adjuvant SOC. (Median Survival: Vehicle_Vehicle 134 days, Vehicle_SOC: 184.5 days, 30 mg/kg aspirin_SOC:190.5, 120 mg/kg aspirin_SOC: 162.5).

Primary resected tumours underwent IHC analysis to determine the effect of low‐dose aspirin on proliferation, necrosis and lymphangiogenesis. As seen in the Her2^+^ models, aspirin did not alter the expression of COX‐2 or changed the extent of tissue necrosis in the TN PDX (Figure 5C). IHC analysis of primary PDX tumours showed no significant decrease (*p* = 0.7326 Kruskal– Wallis) in proliferation (Figure 5D).

Nevertheless, a significant decrease in the expression of VEGF‐D was evident in PDX tumours after 30 mg/kg and 120 mg/kg aspirin (Figure 5E *p* = 0.0320 *N* = 3 Mann–Whitney) but no VEGF‐C expression was seen using IHC. As previously seen in the Her2^+^ setting, neither dose of aspirin altered COX‐2 expression (Figure 5E). The number of lymph vessels did not significantly change within the tumour after pre‐treatment with 30 mg/kg aspirin or 120 mg/kg aspirin daily (Figure 5F) (*P* = 0.2960 *N* = 6 Kruskal–Wallis).

## 4 DISCUSSION

We have established faithful pre‐clinical models of BC subtypes to study the anti‐metastatic, anti‐lymphangiogenic and survival effects of low‐dose aspirin, when delivered prior to tumour resection. Furthermore to investigate the mechanisms responsible for the observed reduction in lymphangiogenesis in HER2+ BC we developed a simple in vitro co‐culutre system of the MSCs and HCC1954.

We first considered the effect of low‐dose aspirin in the Her2^+^ setting. Pre‐treatment with 120 mg/kg aspirin daily caused a significant decrease in primary tumour size (at time of resection) in both MDA‐MB‐231/LM2‐4/H2N and trastuzumab‐resistant HCC1954 Her2^+^ models. Tumours treated with 30 mg/kg aspirin daily, showed reduced tumour size only in the slower growing, less aggressive HCC1954 model. Wu et al. also studied the effect of aspirin on primary tumour growth in the Her2^+^ setting by employing a subcutaneous Her2^+^ SKBR3 model. Animals that received 100 mg/kg aspirin daily for 30 days, showed a significant decrease in primary tumour growth.^26^ Aspirin was shown to exert its effect by inhibiting cell proliferation, regulating cell cycle progression, and inducing apoptosis, through activation of AMP‐activated protein kinase (AMPK) signalling. Previously, Henry et al^27^ investigated the effect of aspirin on the growth of PI3K mutant BC tumours. It was found that mutant PIK3CA‐expressing BC cells had a greater sensitivity to aspirin‐mediated growth suppression than their wild‐type counterparts. The effects of aspirin were ascribed to AMPK activation and autophagy induction. As HCC1954 has a gain in function PI3K (H1047R) mutation, we investigated the induction of autophagy via LC3 cleavage in our model. A significant (twofold) increase in LC3 cleavage in HCC1954 tumours treated with 120 mg/kg aspirin was observed. Therefore, the delay in tumour growth may result from induction of autophagy as well as a reduction in proliferation (as shown previously by a reduced proliferation index). It has further been demonstrated that beta‐catenin activity is a negative regulator of autophagy. Normally beta‐catenin promotes cell survival and inhibits the induction of autophagy.^28^ However, we have shown that beta‐catenin expression in the HCC1954 aspirin‐treated tumours showed a significant upregulation in tumours treated with 120 mg/kg aspirin daily. This contradiction requires further investigation in future studies.

To investigate aspirin's ability to diminish metastasis and improve survival in an in vivo setting, we followed our HCC1954 implanted animals until morbidity. As previously stated, HCC1954 is resistant to trastuzumab due to a gain in function mutation in PI3K (H1047R)^19^ Here, therapeutic resistance was confirmed as no survival benefit was observed following SOC (trastuzumab and paclitaxel) treatment alone. We further demonstrated that animals which received pre‐treatment aspirin (either daily 30 mg/kg or 120 mg/kg aspirin) and subsequent SOC received no significant survival benefit compared to animals that were not pre‐treated with aspirin prior to adjuvant SOC. Despite the observed lack of pre‐treatment aspirin survival benefit in the trastuzumab‐resistant HCC1954 model of Her2^+^ BC, we did observe a delayed time to primary tumour recurrence, and time to metastasis in the MDA‐MB‐231/LM2‐4/H2N Her2^+^ BC model treated with 120 mg/kg aspirin. However, these HER2^+^ cell line models (HCC1954 and MDA) data ostensibly contrast with data from our co‐author's [KB] previous epidemiological analysis of Irish BC patients who received pre‐treatment aspirin. In this study (740 women with stage I‐III BC, taking aspirin for at least 1 year before BC diagnosis^6^) no significant association was demonstrated between aspirin use prior to diagnosis and LN metastasis in Her2^+^ BC. Nevertheless, it is important to consider that there is significant variability in the documented effects of aspirin as analysed across several large‐scale retrospective epidemiological studies. For example, in a Swedish study of 2407 women with Her2^+^ BC, low‐dose aspirin use prior to BC diagnosis was not associated with a decreased risk of recurrence or metastasis either in univariate or covariable‐adjusted analyses.^29^ Moreover, in the same study there was no survival benefit seen in women who had taken aspirin prior to BC diagnosis in the Her2^+^ setting.^28^

**FIGURE 6:**
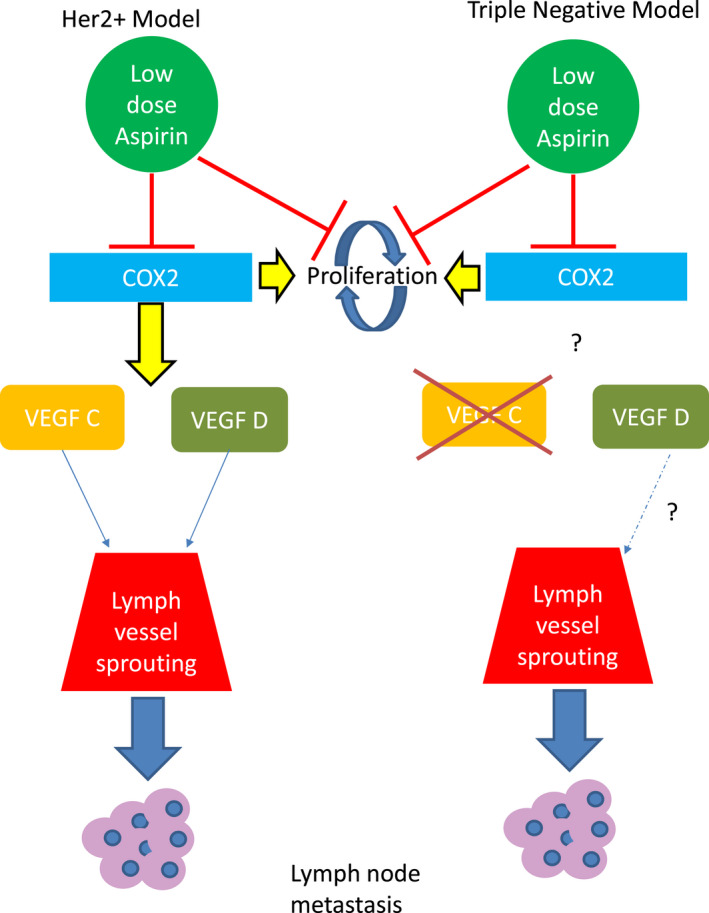
Proposed mechanism of action of low‐dose aspirin in Her2+ and TN breast cancer. (A) Low‐dose aspirin in the HER2^+^ models of BC were shown to inhibit the enzyme COX‐2 and decreases proliferation and furthermore causes a reduction in the expression of lymphangiogenic factors VEGF‐C and D. The reduced expression of the lymphangiogenic factors resulted in significantly lower lymphangiogenesis causing fewer lymph vessels to grow in the primary tumour. The decrease in lymphangiogenesis caused a delay in time to metastasis in the MDA‐MB231/LN2‐4/H2N model of Her2+ BC. (B) In the TNBC model it was shown that aspirin had no effect on proliferation as demonstrated by Ki67. The PDX model also was shown to lack expression of VEGFC making it insensitive to anti‐lymphangiogenic effect of low‐dose aspirin. Low‐dose aspirin does not decrease the number of lymph vessels in the primary tumour

It has been suggested that low‐dose aspirin exerts its effect on the tumour microenvironment and plays a significant role in tumour control and metastasis.^14^, ^15^ Therefore, we next investigated the effect of aspirin on MSCs and HCC1954 in vitro. Low‐dose aspirin abrogated the secretion of VEGF‐C in MSCs alone and in co‐culture with trastuzumab‐resistant HCC1954 cells. Furthermore, the inhibition of VEGF secretion by aspirin significantly diminished the angiogenic potential of the HCC1954 in concert with MSCs. MSCs have been closely associated with tumour progression and metastasis via the promotion of lymphangiogenic properties of lymphatic endothelial cells.^30^, ^31^ Critically, Robering et al. showed that conditioned media from MSC or MSCs co‐culture with lymphatic endothelial cells (LEC) promoted the growth, migration and sprouting of the LECs. They further demonstrated that this effect was partly promoted by secretion of VEGF‐C.^30^ Here, we observed that pre‐treatment aspirin reduces the expression of lymphangiogenic factors (VEGF‐C/‐D) and inhibits lymphangiogenesis in both the Her2^+^ BC models. We therefore hypothesise that the reduction in VEGF‐C and likely VEGF‐D and the subsequent reduction in lymphangiogenesis is due to aspirin's effect on the tumour microenvironment (Figure 6). To further investigate the effect of low‐dose aspirin on HCC1954 angiogenic potential, an angiogenic protein array assay was employed. The addition of 7.5 mM aspirin to cells significantly decreased the expression of urokinase‐type plasminogen activator (UPA) and enhanced the expression of its inhibitor Serpin E1. uPA has previously been shown to be reduced after aspirin treatment in a prosate cancer models in an NF‐kB‐mediated manner.^32^, ^33^ Specifically, Shi et al. showed that uPA secretion was decreased in prosate cells treated with aspirin by transwell assay as well as a concurrent decrease in tumour cell migration and invasion.^33^ It has further been shown this decrease in uPA by aspirin is mediated by inhibition of NF‐kB.^32^ Moreover it has been demonstrated that NF‐kB is a target for aspirin.^34^

Finally, we investigated the effect of low‐dose aspirin in a TNBC PDX model, where treatment with aspirin resulted in significantly reduced tumour sizes as measured by callipers at time of resection. However, there was no observed effect of aspirin pre‐treatment on tumour cell proliferation, which suggests that aspirin mediates tumour growth in the TNBC setting via an alternative mechanism (e.g., by autophagy as discussed above). Furthermore, we showed no reduction in the number of lymphatic vessels in the primary tumours of mice pre‐treated with low‐dose aspirin. Overall our data indicates that aspirin does not effectively reduce lymphangiogenesis and metastatic dissemination in a TBC PDX model of BC that lacks VEGF‐C. Interestingly, tumour or tumour microenvironment cells from our TNBC PDX did not express VEGF‐C^34^ which suggests that lymphangiogenesis is not mediated by VEGF‐C in this model. While BC, and BC PDXs in general, metastasise via the lymphatics and LNs,^35^ it has previously been observed that PDX models can metastasise via the blood.^35^ Our data suggest that PDX HBCX‐12A does not metastasise via the lymphatics, as metastatic lesions were not detectable within the LNs, despite evidence of significant lung dissemination. We have also shown that animals bearing TNBC PDXs which received pre‐treatment aspirin prior to tumour resection and adjuvant SOC therapy (paclitaxel 5 mg/kg once a week for 6 weeks) received no survival benefit compared to animals that were not pre‐treated with aspirin prior to adjuvant SOC. In a retrospective analysis, Williams et al. studied the survival of 1113 women with BC which included TNBC. It was shown that aspirin use prior to diagnosis was associated with decreased overall survival compared to women not taking aspirin when adjusted for SOC therapy.^36^ This negative association was also found in several other clinical TNBC studies.^29^, ^37^ Smeda et al. also recently reported the effects of long‐term use of low‐dose aspirin (120 mg/kg) in a syngeneic mouse model of TNBC (4 T1). As demonstrated here, pre‐treatment aspirin had no effect on survival or overall metastatic burden.^38^

## 5 STUDY LIMITATIONS

One limitation of the current study relates to the use of the metastatic triple negative PDX model; while the model robustly metastasised to the lungs of the mice, it was shown (by post hoc IHC analysis) that the PDX lacked expression of VEGFC and lymphatic‐mediated metastasis. In future studies, models should be screened (a priori) for the expression of the lymphangiogenic factors to ensure optimal model selection. Furthermore, we recognise that future pre‐treatment aspirin mechanistic studies employing the MDA‐MB‐231/LM2‐4/H2N _Luc2 should incorporate post resection SOC therapy.

## 6 CONCLUSION

Overall, we have established faithful pre‐clinical models to study the anti‐metastatic, anti‐ lymphangiogenic and survival effects of low‐dose aspirin, when delivered prior to tumour resection in BC subtypes. Aspirin delayed time to metastasis and inhibited lymphangiogenesis in a Her2^+^ BC model (MDA‐MB‐231/LM2‐4/H2N_Luc2). Autophagy induction as demonstrated in the HER2^+^ HCC1954 primary tumours may be responsible for the observed tumour delay. We have further demonstrated that low‐dose aspirin inhibits the (lymph)angiogenic process in HER2^+^ positive tumours and abrogates the secretion of VEGF‐C from MSCs in vitro. Moreover, low‐dose aspirin alters the secretome of trastuzumab‐resistant HCC1954 tumour cells. Overall, we observed that a chemo‐preventative regimen of low‐dose aspirin when delivered in the presence of adjuvant SOC chemotherapy did not overcome therapy resistance in the HER 2^+^ trastuzumab‐resistant HCC1954 Her2 and also did not increase survival when compared with adjuvant SOC chemotherapy alone (HCC1954 model). Moreover, employing a PDX model of TNBC we have further shown that while low‐dose aspirin can delay primary tumour growth, it does not reduce the expression of lymphangiogenic factors nor significantly impact lymph vessel outgrowth. Our data also suggest that low‐dose aspirin pre‐treatment in the TNBC setting does not increase survival compared to SOC alone. These results align with several clinical studies of Her2^+^ and TNBC where pre‐treatment aspirin was not found to provide any significant survival benefit to BC patients.^6^, ^29^, ^39^, ^40^

Overall, the future for aspirin as a preventative treatment in Her2^+^ and TNBC remains uncertain. Retrospective epidemiological studies have failed to provide clarity on its role a chemo‐preventative agent in BC.^4^, ^6^, ^29^, ^36^, ^41^ Large‐scale, long‐term, well designed prospective clinical trials are needed to critically understand and re‐evaluate the role of aspirin as a chemo‐preventative in this setting.

## CONFLICT OF INTEREST

All other authors declare no conflict of interest. The funders had no role in the design of the study; in the collection, analyses or interpretation of data; in the writing of the manuscript, or in the decision to publish the results.

## AUTHOR CONTRIBUTIONS

Conceptualization, WMG, RMD and ATB; methodology, WMG, SC, RS, PL, RK, KB, RMD and ATB; formal analysis, ISM, SK, LPS, SD, BM, CI, PL, EC, EM, DPOC; investigation, ISM, LPS, SD, ACOF, KC, AL, BM, PL, SC, DPOC; resources, WMG, EM, KB, RMD, ATB; data curation, ISM; writing—original draft preparation, ISM SK ATB; writing—review and editing, ISM LPS ACOF, KC, SC, KB, and A.T.B; supervision, W.M.G, A.T.B; funding acquisition, KB, WMG, ATB. All authors have read and agreed to the published version of the manuscript.

## ETHICAL APPROVAL STATEMENT

In vivo studies were performed at University College Dublin (UCD) and were approved by UCD Animal Research Ethics Committee (AREC). All work was performed according to local rules and regulations and under Health Products Regulatory Authority HPRA licence number AE18982/P039.

## Supporting information


Figures S1−S4

Data S1

## Data Availability

The data that supports the findings of this study are available in the supplementary material of this article.
