# Supplementary material for: Implementing subtype‐specific pre‐clinical models of breast cancer to study pre‐treatment aspirin effects
Source: Cancer Med. 2022 Apr 17;11(20):3820–36. doi: 10.1002/cam4.4756 (PMC9582689; doi:10.1002/cam4.4756)

Figure S1: Pharmacokinetic analyses of mouse blood plasma after treatment with low doses of aspirin to establish clinically relevant dosing regimen in mice.

A

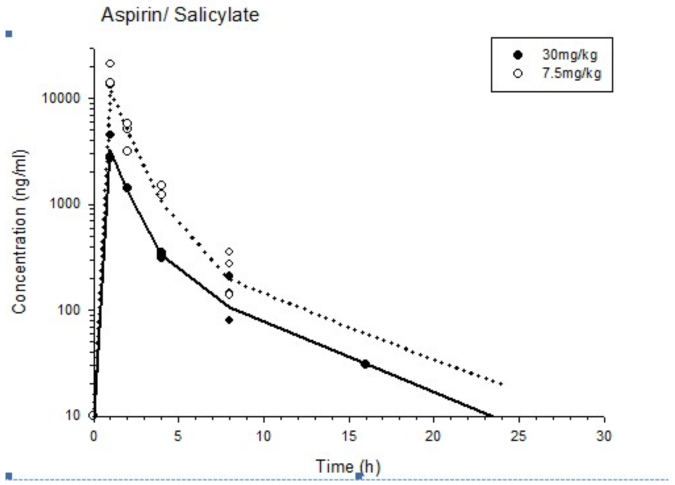

B

	7.5mg/kg	30mg/kg
A (ug/ml)	8.19	28.8
B (ug/ml)	344	592
Kela (h^{-1})	1.00	0.92
Kelb (h^{-1})	0.15	0.14
AUC ug h /ml	7.29	24.3

C

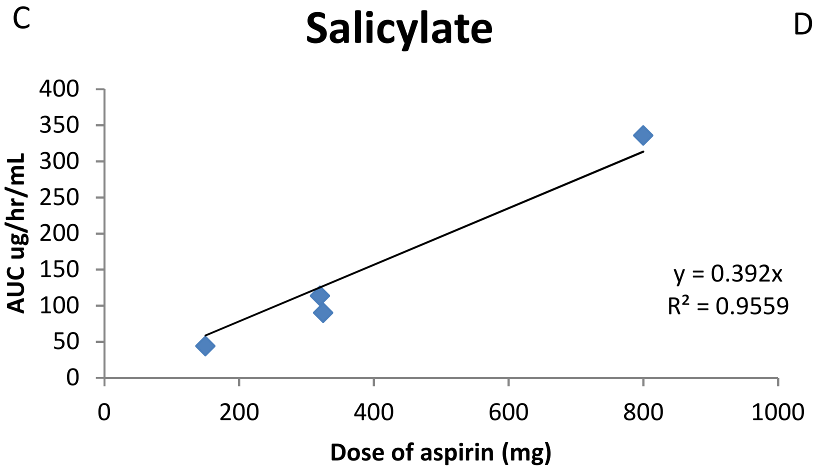

D

	mg	AUC		mg/kg	AUC
Human	75	24.4	Mouse	30	24.3
Human	300	117.6	Mouse	120	117.5

E

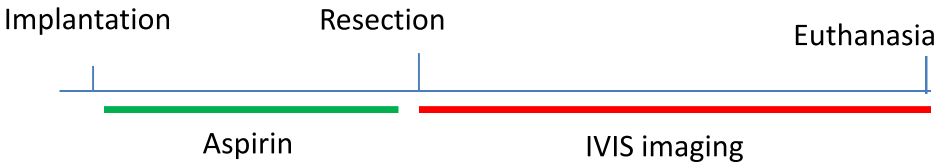

F

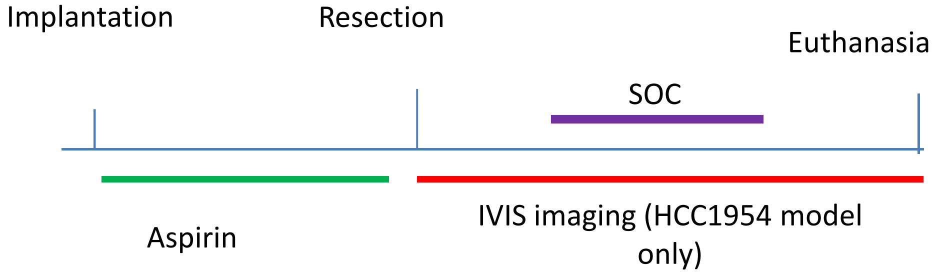

Figure S2. Pre-treatment of low dose aspirin reduces the growth of the primary tumor, delays reoccurrence metastasis in the MDA-MB 231/LN2-4/H2N Her2+model of breast banker as shown by bioluminescence

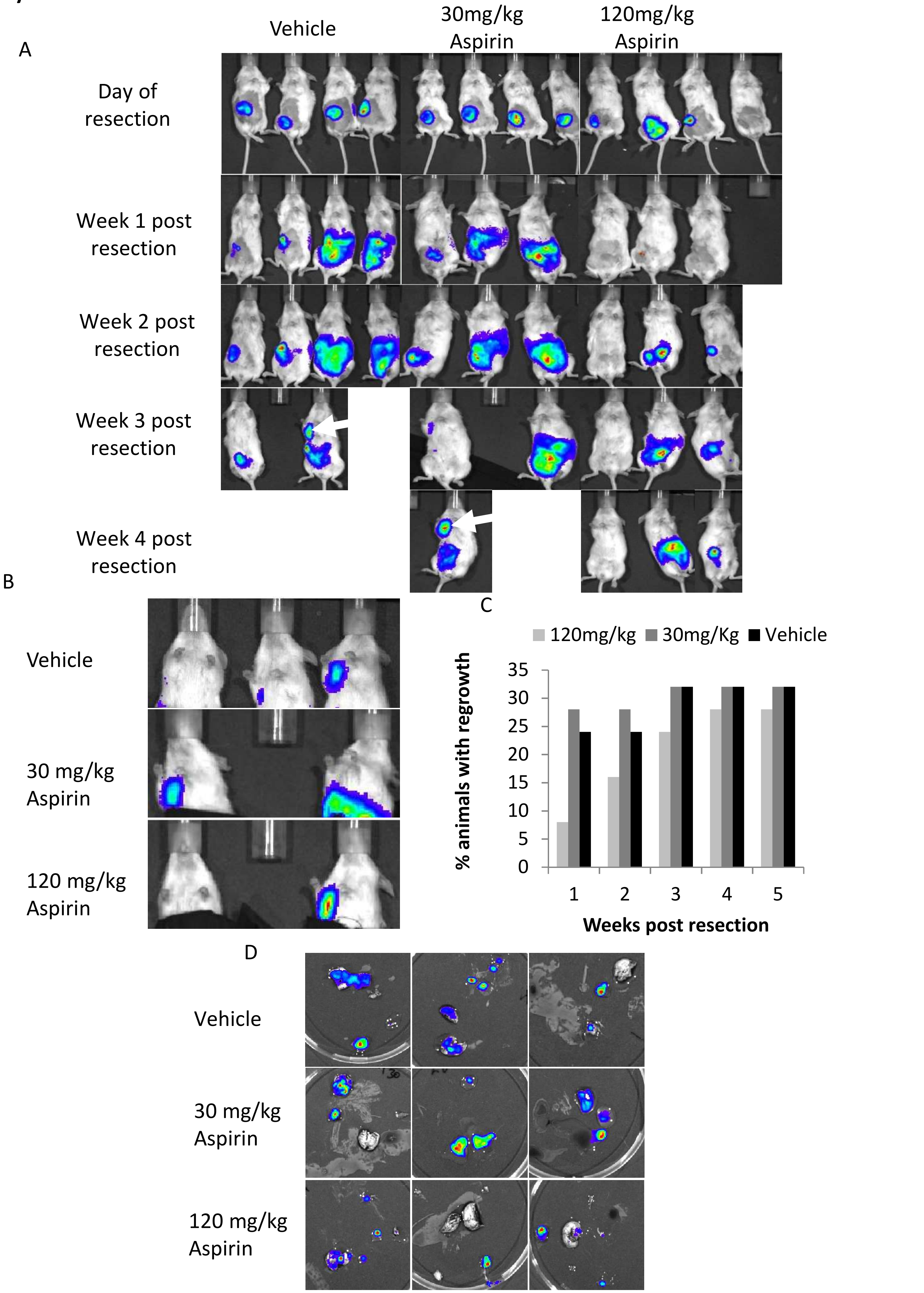

Figure S3. Confirmation of the resistance of HCC1954 to HER2 standard of care therapy

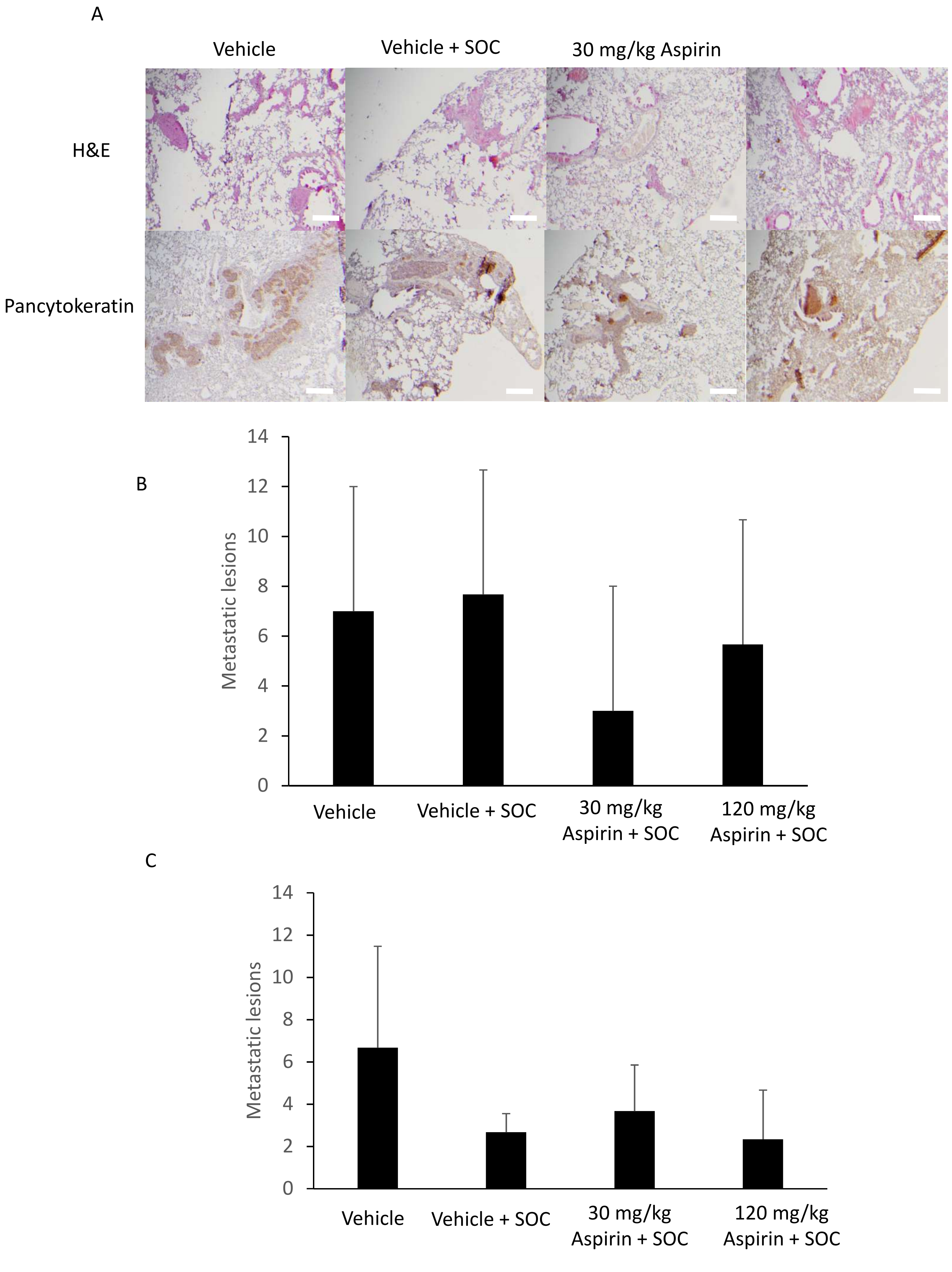

Figure S4: Triple-negative PDX tumor TN173 metastasises via the blood and not by the lymphatics.

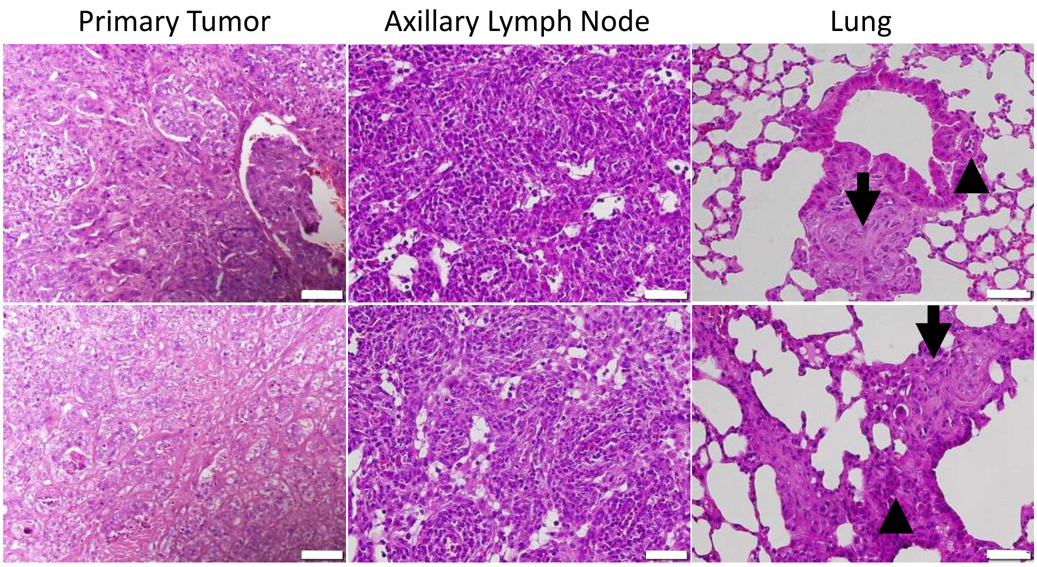

Supplement: Supplementary file 1 — Figures S1−S4 [file CAM4-11-3820-s002.pdf]
