# Supplementary material for: Implementing subtype‐specific pre‐clinical models of breast cancer to study pre‐treatment aspirin effects
Source: Cancer Med. 2022 Apr 17;11(20):3820–36. doi: 10.1002/cam4.4756 (PMC9582689; doi:10.1002/cam4.4756)
Supplement: Supplementary file 2 — Data S1 [file CAM4-11-3820-s001.docx]

**Supplementary Methods:**

*S1.1 Pharmacokinetic analyses to establish clinically relevant dosing regimen for aspirin in mice*

42 female NOD/SCID mice were divided into 2 groups (N=21) given a single dose of aspirin (n=21 7.5mg/kg; n=21 30mg/kg) orally. At scheduled time points (0.5, 1, 2, 4, 8, 16 and 24 h) 3 mice from each group were euthanized and had their blood drawn via cardiac puncture. The blood was collected into heparinized tube to inhibit clotting. The blood was centrifuged for 15 min at 3000g to collect plasma. Plasma samples (in triplicate) were analysed on a triple quadrupole mass spectrometer utilizing salicylic acid as a control reference. The lower limit of detection was 20ng/mL. Area under the cover analysis of the plasma curves was performed to determine the kinetic of salicylate absorption in the plasma. Utilizing human values obtained from the literature ^44-46^ a correlation was obtained to determine the equivalent aspirin concentration in mice.

*S1.2 Cell culture*

Human BC cell lines HCC-1954 (HCC1954: CVCL_1259 Naturally HER2 overexpressing) and MDA-MB 231/LN2-4/H2N (CVCL_0062) were grown in RPMI 1640 (Sigma), supplemented with 10% (v/v) foetal bovine serum (FBS, Sigma) and L-glutamine (Sigma), in 5% CO2 at 37°C. Cells were used at passages 4-6. Following ethical approval and informed consent, Adult human Mesenchymal Stem Cells (MSCs) were isolated from the iliac crest of healthy volunteers. The differentiation capacity and cell antigen profile of the MSCs was confirmed prior to use. MSCs were routinely cultured in α-Minimum Essential Medium (αMEM) supplemented with serotyped 10% FBS, Pen/Strep and 1 ng/ml Fibroblast Growth Factor (FGF). Cells were used at passages 4-6.

Cells were regularly checked for the presence of mycoplasma and IMPACT 1 tested for the presence of mouse pathogens before implantation into mice.

*S1.3 Lentiviral transduction of* HCC1954 *cell line*

Lentiviral transduction of HCC1954 cells with the luciferase gene was performed. All virus work was conducted according to Environmental Protection Agency (EPA) guidelines. Briefly, HEK 239T cells were grown in RPMI 1640 media, supplemented with 10% (v/v) FBS and L-Glutamine, and seeded in a T25 flask one day prior to transfection so that they were 80% confluent. A transfection mix was prepared containing: 30μl Opti-MEM I (Invitrogen), 9μl Gene juice (transfection reagent, Novagen), 1μg plasmid DNA (plenti6-luc 2), 1μg packaging vector (psPAX2) and 0.8μg envelop vector (pMD2.G). This was added to 3ml of RPMI 1640 media, and incubated with 239T cells overnight. The media was replaced with 4ml of fresh RPMI media, and incubated for an additional 48h. The media was then removed and filtered through a 0.45μm binding filter (Millipore), and 8μg/ml polybrene (infection reagent, Sigma) was added. This transduction medium was added to the target cells, and left to incubate for 24h. The media was then refreshed, and the cells passaged as required. After 3 passages cells were removed from the virus room treated with Blasticidin S for antibiotic selection.

*S1.4 Establishment of MDA-MB-231 LM-2/4 H2N cell line xenografts*

Previously the MDA-MB-231 LM-2/4 H2N was created by Munoz et al. ^47^. Briefly, parental MDA-MB-231 Erb2 overexpressing cells were orthotopically implanted into mice. Tumors were resected as reported here and metastases were allowed to develop. These metastases were then removed, disaggregated and re-implanted into a new cohort of mice. This serial passaging was repeated 4 times to create a highly aggressive variant that metastasized reliably to the lungs and lymph nodes of mice after surgical resection of the primary tumor.

*S1.5 Bioluminescence Imaging (BLI)*

BLI was employed to monitor metastatic dissemination. Mice were imaged weekly until tumors reached 15mm in any give dimension then euthanized and organs harvested. *Ex vivo* BLI was performed to detect level of metastatic spread. Imaging was performed with an IVIS Spectrum (Perkin Elmer). 15min prior to imaging mice were injected IP with 150mg/kg Luciferin (Perkin Elmer). A 1 sec reference image was then taken with binning set to 4 and F-stop 1. Images analysis was performed using Living Image software (V4.3.1, Perkin Elmer).

*S1.6 H&E staining*

All orthotopic tumors were resected, rinsed twice in D-PBS and fixed in 4% formaldehyde for 48 h. Tissues were embedded in paraffin and 5 μm thick sections were cut. Routine haematoxylin and eosin (H&E) staining was performed to facilitate histological evaluation. H&E staining was performed using a Leica autostainer XL (Leica, Wetzlar, Germany). Slides were deparaffinised and immersed in haematoxylin (10min), washed 3 times in water then immersed in eosin for 90 seconds. Sections were washed again 3 times then dehydrated and cover slipped**.**

*S1.8 Conditioned medium collection*

Indirect co-culture of cells was performed using polycarbonate Transwell® inserts (Corning™, 0.4µm pores) in a 6-well plate format. MSCs (2.5 x 105) were seeded into the wells and HCC-1954 breast cancer cells (1.5 x 105) were seeded into the inserts. Each population was also cultured individually as a control. Following overnight incubation cells were treated with Aspirin (2.5 or 7.5 mM) or diluent control (1% DMSO). Cell Conditioned media (CM) was harvested after 24 hours and centrifuged at 2000 x g for 5 mins to remove cell debris. CM was then stored at -80°C until further use..

*S1.9 Western Blot analysis of LC3*

Protein was extracted form primary resected HCC1954 tumours (3 tumours per group) that were treated +/- aspirin. Total protein was adjusted to equal amounts, and 25ug of protein mixtures were separated via 12% SDS-PAGE and transferred to polyvinylidene difluoride (PVDF) membranes. After the transfer, non-specific binding sites of the membranes were blocked for 1 h at room temperature in PBS (pH 7.4) containing 5% (wt/vol) non-fat dry milk and then incubated with primary antibody rabbit α-LC3-I/II (1:1000 Abcam ab48394), at 4 °C overnight. The membranes were probed with an mouse anti-tubulin antibody (1:1000 Abcam ab7291) to control protein loading, and then incubated for 1 h at room temperature with HRP-conjugated secondary antibodies (1:10000). Densitometry measurements were performed with Image J. The band intensities were semi-quantified via densitometry analysis and relative protein expression was normalised to tubulin and compared with the control group.
